# Structural Characterization, Dielectric Properties and Energy Storage Performance of Co-Electrospun PVA and P(VDF-HFP) Nanofibers

**DOI:** 10.3390/ijms27062622

**Published:** 2026-03-13

**Authors:** Kunlawan Hirunchulha, Suphita Chaipo, Ponkrit Itsaradamkoeng, Thanatat Rodprapai, Chatchai Putson

**Affiliations:** Materials Physics Laboratory, Division of Physical Science, Faculty of Science, Prince of Songkla University, Hat Yai, Songkhla 90110, Thailand; 6710230024@psu.ac.th (K.H.); beamsuphita@gmail.com (S.C.); game.ponkrit@gmail.com (P.I.); rodprapaiice@gmail.com (T.R.)

**Keywords:** PVA/P(VDF-HFP) nanofibers, co-electrospun, electrospinning, energy storage density, dielectric properties

## Abstract

In this work, biodegradable poly(vinyl alcohol) (PVA) and ferroelectric poly(vinylidene fluoride-co-hexafluoropropylene) (P(VDF-HFP)) nanofibers were successfully fabricated via co-electrospinning. The morphology and microstructure of co-electrospun PVA/P(VDF-HFP) nanofibers were analyzed, demonstrating that P(VDF-HFP) incorporation significantly affected fiber diameter and phase distribution. These structural features altered the fiber diameter and surface area of the co-electrospun system, thereby affecting interfacial polarization and the resulting dielectric and energy storage performance. As a result, the dielectric constant of the PVA/P(VDF-HFP) nanofibers (M1) was enhanced by up to 1.8 times compared with pure PVA nanofibers (M0), owing to interfacial polarization arising from increased surface charge accumulation at the PVA/P(VDF-HFP) interfaces. Meanwhile, dielectric loss and electrical conductivity were effectively controlled, indicating improved electrical stability of the co-electrospun system. Furthermore, ferroelectric and energy storage analyses revealed that appropriate incorporation of P(VDF-HFP) and phase distribution significantly enhanced polarization and energy storage performance. The energy storage density increased from 0.83 to 3.21 mJ cm^−3^ at 20 MV m^−1^, corresponding to an improvement of 287% while maintaining a high energy efficiency of approximately 90%. Owing to their favorable dielectric properties, mechanical flexibility, and environmental compatibility, the co-electrospun PVA/P(VDF-HFP) nanofibers demonstrate great potential for low-field wearable and biomedical energy storage devices.

## 1. Introduction

Polymer dielectric materials have attracted extensive attention over the past several decades owing to their outstanding properties, including lightweight nature, high mechanical flexibility, facile processability, low cost and excellent electrical properties [1,2]. In addition, polymer dielectrics exhibit good tunable properties, enabling their widespread application in modern electronic and biomedical devices such as biosensors, wearable electronics, and flexible energy storage systems [3]. As a result, achieving a balance between mechanical flexibility and dielectric performance has become a critical challenge in the development of polymer dielectrics. Therefore, polymer-based materials with high dielectric permittivity, low dielectric loss, and preserved structural flexibility have been intensively investigated using various fabrication strategies, including nanocomposite preparation, polymer blending, and electrospinning techniques [4,5,6].

Electrospinning represents a cost-effective and straightforward technique for fabricating nanofibrous materials, enabling the formation of highly porous structures with large specific surface areas and fiber diameters spanning from the nanometer to micrometer scale [7]. Such fibrous structures generate abundant interfacial regions. These interfacial regions facilitate Maxwell–Wagner–Sillars interfacial polarization, thereby enhancing charge accumulation [8]. In heterogeneous dielectric materials, interfacial polarization arises from the accumulation of charges at the interfaces between regions with different electrical properties, including the functional interlayers in sandwich structures. Moreover, fiber-based structures enable effective redistribution of local electric fields and reduce field concentration, which helps to suppress premature dielectric breakdown and improve operational stability. Recently, increasing attention has been given to fabricating dielectric materials in fiber form, as the enlarged specific surface area enhances interfacial charge accumulation, thereby improving the overall dielectric performance [9].

Among polymer dielectrics, poly(vinyl alcohol) (PVA) has attracted considerable interest due to its high flexibility, environmental friendliness, and biodegradability [10]. In addition, PVA exhibits low dielectric loss, which is an important characteristic for dielectric applications. Previous studies have reported the use of PVA as a dielectric matrix through the incorporation of dopants or functional fillers to enhance dielectric permittivity and improve electrical performance [11,12]. Nanofibrous structures offer a high specific surface area that promotes interfacial polarization. Nevertheless, the intrinsically low dielectric permittivity and hydrophobic nature of PVA limit its suitability for energy storage applications. Accordingly, engineering PVA into nanofiber-based nanocomposites can effectively address these shortcomings and enhance dielectric and energy storage performance. Wenjie Yu et al. developed stretchable MXene/PVA fibers by electrospinning for wearable supercapacitors with good mechanical stability. PVA was used for its flexibility, abundant –OH groups for strong bonding with MXene, and improved fiber processability [13]. However, the high conductivity of MXene/PVA fibers is an electrical limitation, which can create leakage pathways and increase energy loss, thereby reducing dielectric performance and limiting the achievable energy storage capability. In addition, the dielectric properties are highly dependent on the uniform dispersion of MXene within the PVA matrix; aggregation of nanosheets may cause local electric field concentration and deteriorate dielectric stability. Therefore, combining PVA with polymers possessing strong electric dipoles, such as those in the PVDF family, represents a promising strategy to enhance dielectric properties and improve energy storage performance. Nevertheless, studies examining the effects of fiber dispersion and hybridization in mixed-fiber membranes, as well as their associated morphological characteristics, on dielectric polarization behavior and energy storage performance remain limited. A deeper understanding of these relationships is therefore essential for optimizing the microstructure of fibrous polymer systems and improving their dielectric and capacitive energy storage capabilities.

Meanwhile, poly(vinylidene fluoride-co-hexafluoropropylene) [P(VDF-HFP)] is a promising dielectric and ferroelectric polymer owing to its high polarization capability and excellent chemical stability [14]. Poly(vinylidene fluoride) (PVDF) is a semi-crystalline polymer that can crystallize into several polymorphic phases, namely α, β, γ, δ, and ε. Among these, the β phase is the most electroactive form due to its all-trans (TTTT) planar zigzag conformation, which enables strong dipole alignment along the polymer chain. This molecular arrangement is responsible for its superior piezoelectric, ferroelectric, and dielectric properties compared to the other phases [15,16].

The formation and stabilization of the β phase can be significantly enhanced through electrospinning. Numerous studies have demonstrated that the β-phase can be enhanced through various approaches, such as chemical doping, mechanical stretching, and electrospinning, where the strong electric field and elongational forces during fiber formation promote molecular chain alignment.

The combination of P(VDF-HFP) and PVA represents an effective strategy to integrate complementary electrical and mechanical functionalities within a single system. In this synergistic configuration, P(VDF-HFP) provides strong dipolar polarization associated with its electroactive phases, while PVA contributes intrinsic polarity, mechanical flexibility, and structural regulation that can mitigate local electric field concentration, thereby enhancing dielectric stability and hydrophobic properties.

Therefore, this work focuses on the development of polymer-based dielectric fibrous architectures fabricated via co-electrospinning. Co-electrospun PVA and P(VDF-HFP) fibers are simultaneously deposited with systematically varied composition ratios, from pristine PVA to structures containing progressively higher fractions of P(VDF-HFP) fibers. The morphology and microstructure of co-electrospun PVA/P(VDF-HFP) nanofibers were analyzed, demonstrating that P(VDF-HFP) incorporation significantly affected fiber diameter and phase distribution. The findings are expected to clarify the relationship between fiber architecture, phase crystallinity, dielectric behavior, and energy storage performance, offering insights for the design of flexible electronic and energy storage applications.

## 2. Results and Discussion

### 2.1. Scanning Electron Microscope (SEM)

**Figure 1:**
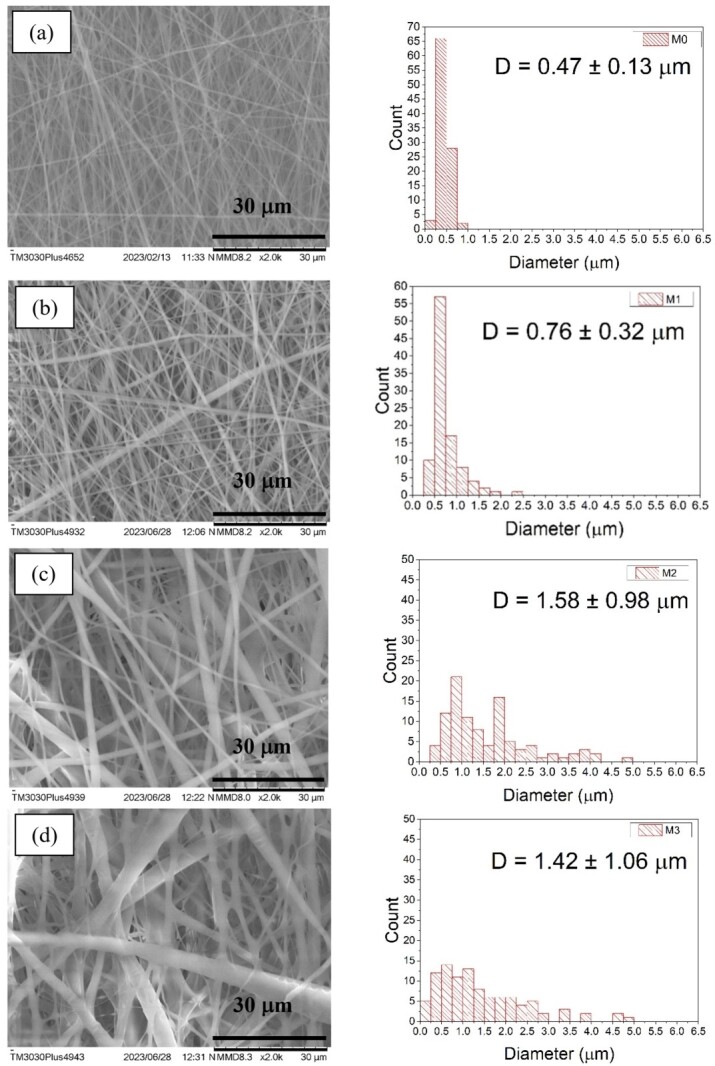

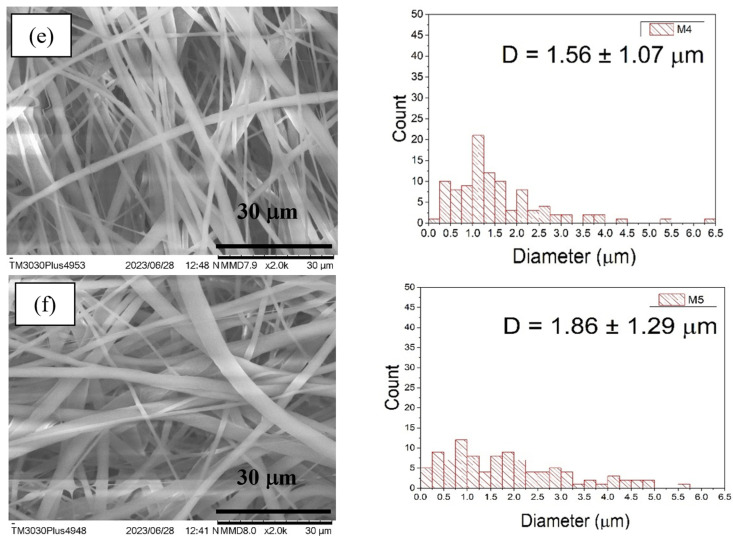
SEM micrographs and corresponding fiber diameter distribution histograms of the electrospun samples: (**a**) M0, (**b**) M1, (**c**) M2, (**d**) M3, (**e**) M4, and (**f**) M5, a PVA flow rate was fixed at 1.0 mL h^−1^ while the flow rate of the P(VDF-HFP) solution was varied at 1.0, 2.0, 3.0, 4.0, and 5.0 mL h^−1^ for M1–M5, respectively.

The co-electrospun fibers were fabricated via the electrospinning technique by varying the flow rate of the P(VDF-HFP) solution, while the PVA flow rate was kept constant. The structural characteristics of the co-electrospun fibers were controlled by adjusting the P(VDF-HFP) flow rate while maintaining an identical total deposited volume for each sample. As shown in Figure 1a–f, the pure PVA fibers (M0) exhibited the smallest average diameter. Overall, the average fiber diameter increased with increasing P(VDF-HFP) flow rate. Furthermore, the standard deviation (SD) values of the co-electrospun fibers were higher than those of the pure PVA fibers. The smaller SD value observed for M0 indicates a more uniform fiber diameter distribution, whereas the larger SD values for M1–M5 suggest a broader diameter distribution and increased morphological variability at higher P(VDF-HFP) flow rates. Co-electrospinning involves the simultaneous electrospinning of PVA and P(VDF-HFP) solutions from separate syringes, resulting in two types of fibers deposited within the same membrane rather than forming bicomponent fibers. Therefore, the interfaces discussed in this work correspond to the contact regions between adjacent PVA and P(VDF-HFP) fibers within the fibrous network.

### 2.2. Water Contact Angle

**Figure 2:**
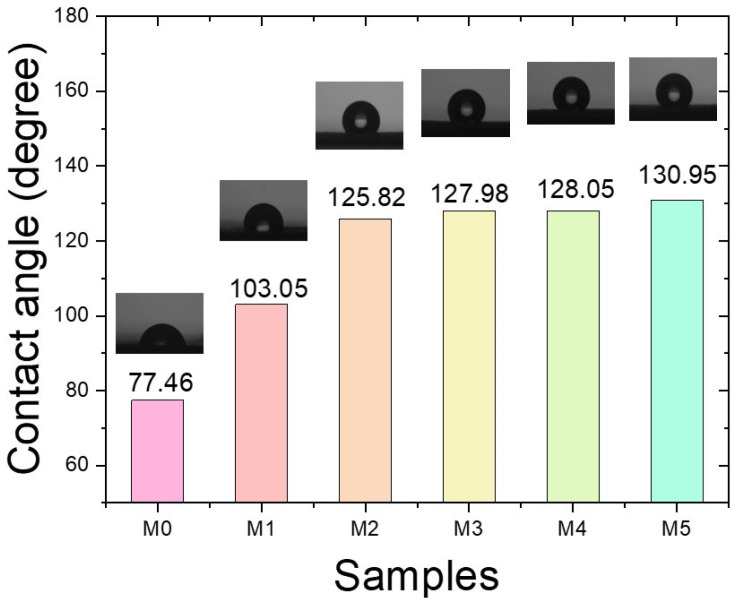
Contact angle for water droplets on co-electrospun fiber membranes at different ratios of P(VDF-HFP).

The wetting characteristics of the co-electrospun fiber membranes were evaluated using static water contact angle measurements with a droplet volume of 3 μL. As shown in Figure 2, the pure PVA nanofiber membrane (M0) exhibited hydrophilic behavior, with a contact angle of 77.46°, consistent with the intrinsic hydrophilicity and water solubility of PVA [17]. In contrast, the contact angles of the co-electrospun membranes (M1–M5) increased progressively with increasing P(VDF-HFP) flow rate. All co-electrospun samples exhibited contact angles greater than 90°, indicating hydrophobic surface characteristics. Among them, M5 demonstrated the highest hydrophobicity, with a contact angle of 130.95°, comparable to previously reported values for electrospun P(VDF-HFP) fiber membranes [18].

The wetting behavior can be interpreted using classical wetting models, namely the Wenzel and Cassie–Baxter models [19,20]. For the PVA nanofiber membrane (M0), the relatively low contact angle suggests that water readily penetrates the surface asperities, corresponding to the Wenzel wetting regime where the liquid completely wets the rough surface. In contrast, the co-electrospun membranes containing hydrophobic P(VDF-HFP) are better described by the Cassie–Baxter regime. In this state, air pockets are trapped within the inter-fiber voids, forming a composite solid–liquid–air interface that increases the apparent contact angle. When the liquid has low affinity for the solid surface, increased roughness further amplifies hydrophobicity under the Cassie–Baxter condition.

Furthermore, it is important to distinguish between the intrinsic contact angle, which depends on the surface chemistry of the material, and the apparent contact angle, which is influenced by surface roughness and microstructure. SEM observations (Figure 1) indicate that increasing the P(VDF-HFP) flow rate leads to larger fiber diameters and a rougher membrane morphology. This hierarchical surface structure promotes air entrapment and enhances the apparent contact angle beyond the intrinsic hydrophobicity of P(VDF-HFP). Consequently, the combined effects of low surface energy and increased surface roughness contribute synergistically to the significant increase in contact angle observed for the co-electrospun membranes compared to the pure PVA nanofiber membrane.

Moisture absorption in PVA-based materials can indeed influence electrical conductivity due to water-induced ionic conduction. As P(VDF-HFP) content increases, the membrane becomes more hydrophobic, as confirmed by the increase in the water contact angle from 77.46° for M0 to 130.95° for M5. This increased hydrophobicity likely reduces water uptake in the membrane, which in turn may suppress water-driven ionic conduction in the PVA phase. Therefore, the reduced moisture absorption with higher P(VDF-HFP) content could contribute to the observed decrease in electrical conductivity.

### 2.3. X-Ray Diffraction (XRD) Analysis 

**Figure 3:**
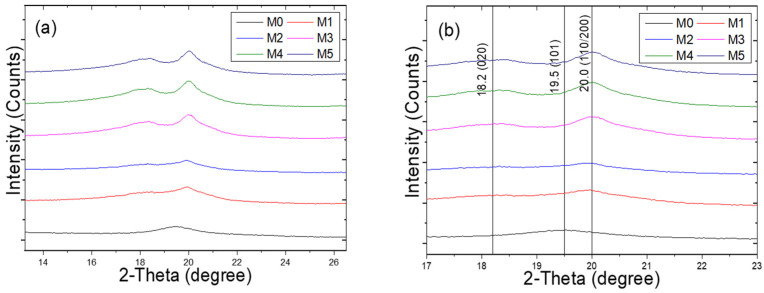
XRD patterns of PVA and P(VDF-HFP) co-electrospun fiber membranes recorded in the 2θ ranges of (**a**) 13–27° and (**b**) 17–23° (enlarged view).

The XRD patterns of the PVA and P(VDF-HFP) co-electrospun fiber membranes are presented in Figure 3. The pure PVA fibers exhibit a single characteristic diffraction peak at 2θ ≈ 19.5°, corresponding to the (101) crystallographic plane [21,22]. Upon incorporation of P(VDF-HFP), the intensity of this peak gradually decreases with increasing P(VDF-HFP) flow rate. Meanwhile, additional diffraction peaks appear at 2θ ≈ 18.2° and 2θ ≈ 20.0°, and their intensities progressively increase as the P(VDF-HFP) flow rate increases. These peaks are assigned to the (020) plane of the α-phase and the overlapping (110)/(200) planes of the β-phase of P(VDF-HFP), respectively [23,24], indicating the coexistence of α and β crystalline phases in the co-electrospun fiber membranes.

**Figure 4:**
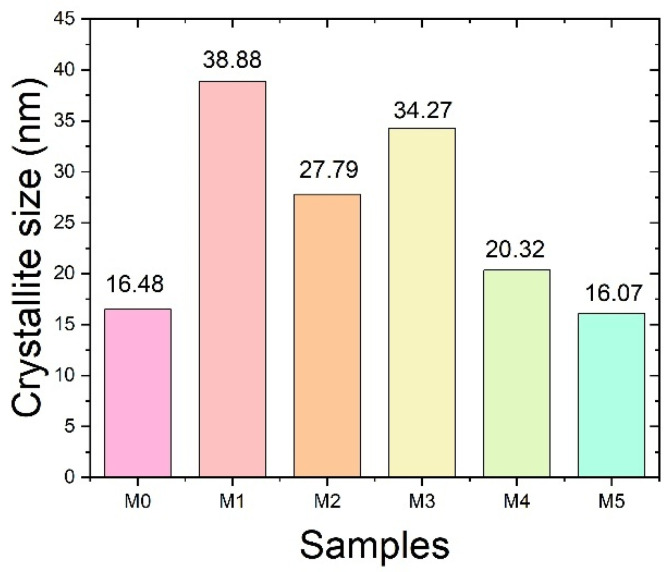
Crystallite size of PVA/P(VDF-HFP) co-electrospun fiber membranes determined from X-ray diffraction (XRD) data using the Scherrer equation.

The crystallite size values, calculated using the Scherrer equation, are presented in Figure 4. The crystallite size increased from 16.48 nm for M0 to 38.88 nm for M1, suggesting enhanced crystal growth at moderate P(VDF-HFP) flow rates. However, further increasing the P(VDF-HFP) flow rate resulted in a decrease in crystallite size to 27.79 nm (M2), 34.27 nm (M3), 20.32 nm (M4), and 16.07 nm (M5). This reduction observed at higher flow rates may be associated with intensified jet stretching and rapid solidification during electrospinning, which can limit crystal growth. The variation in crystallite size suggests a competitive interplay between crystalline ordering and jet-induced stretching effects as the P(VDF-HFP) flow rate increases. Overall, these findings indicate that the crystalline structure and crystallite size distribution of the co-electrospun membranes are strongly influenced by the P(VDF-HFP) flow rate.

### 2.4. Fourier Transform Infrared Spectrophotometer (FTIR)

**Figure 5:**
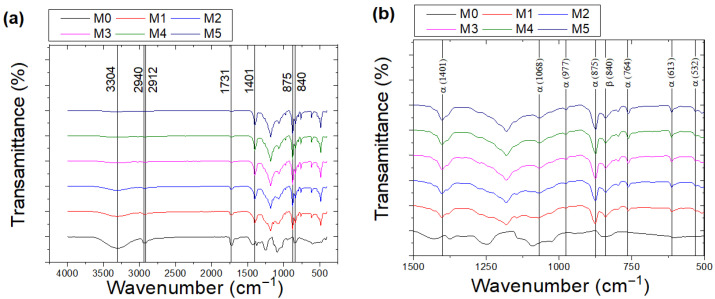
FT-IR spectrum of PVA and P(VDF-HFP) co-electrospun fibers at (**a**) 400–4000 cm^−1^ and (**b**) 500–1500 cm^−1^.

Figure 5 presents the FTIR spectra of PVA and P(VDF-HFP) co-electrospun fiber membranes recorded in transmission mode. The characteristic absorption bands of pure PVA nanofibers are observed at 3304, 2941, 2912, and 1731 cm^−1^, corresponding to O–H stretching, C–H stretching, CH_2_ symmetric stretching, and C=O carbonyl stretching vibrations, respectively [25,26].

For P(VDF-HFP), prominent bands located at approximately 1401, 875, and 840 cm^−1^ are attributed to CF_2_ symmetric stretching and CF_3_ rocking vibrations [27]. With increasing P(VDF-HFP) flow rate, the intensity of PVA-related bands gradually decreases, while the P(VDF-HFP)-associated bands become more pronounced, indicating a progressive change in the relative composition of the co-electrospun membranes.

**Figure 6:**
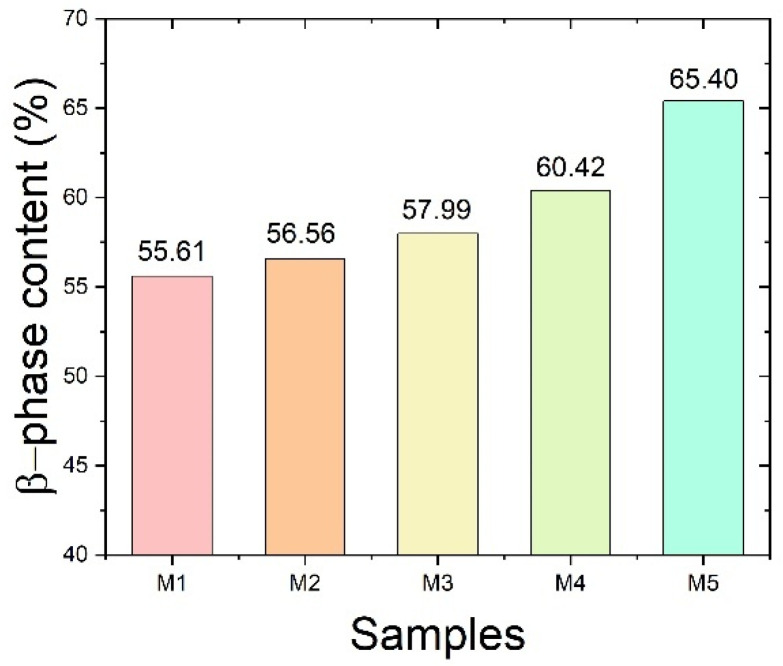
β-phase content of PVA/P(VDF-HFP) co-electrospun fiber membranes.

The β-phase fraction was quantitatively evaluated using the characteristic absorption bands at 764 and 840 cm^−1^, corresponding to the α- and β-phases of P(VDF-HFP), respectively [28]. The relative fraction, F(β), was calculated from the absorbance values of these bands. The 840 cm^−1^ band is attributed to the all-trans (TTTT) chain conformation, whereas the 764 cm^−1^ band corresponds to the TGTG′ conformation of the α-phase. Although the 764 cm^−1^ and 840 cm^−1^ bands may partially overlap with contributions from other phases, the increasing intensity of the 840 cm^−1^ band with increasing P(VDF-HFP) flow rate is consistent with the enhanced β-phase diffraction peak observed in the XRD patterns in Figure 3b. This agreement further confirms the assignment of the 840 cm^−1^ band to the β-phase. As shown in Figure 6, F(β) increases progressively with increasing P(VDF-HFP) flow rate. This trend may result from the combined effect of higher P(VDF-HFP) flow rate and elongational forces during electrospinning, which promote molecular chain alignment along the fiber axis and stabilize the all-trans conformation. In addition, a higher solution flow rate introduces a larger amount of solvent into the electrospinning jet, which prolongs the solvent evaporation time and maintains higher chain mobility during fiber formation. This extended semi-wet state allows the electric field and stretching forces to more effectively orient the polymer chains, thereby facilitating the formation and stabilization of the β-phase.

### 2.5. Dielectric Behavior

**Figure 7:**
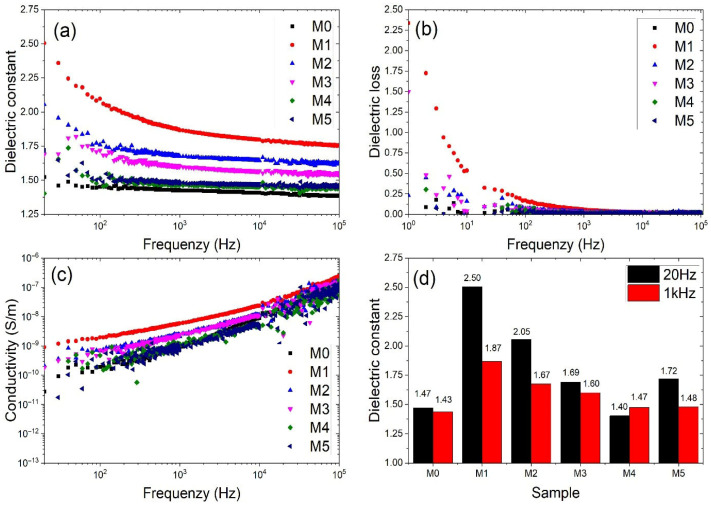
Frequency-dependent dielectric properties and electrical conductivity of PVA/P(VDF-HFP) co-electrospun fiber membranes: (**a**) dielectric constant, (**b**) dielectric loss, (**c**) electrical conductivity; and (**d**) dielectric constant of co-electrospun fiber membranes at 20 Hz and 1 kHz.

The dielectric constant (ε_r_) of the PVA and P(VDF-HFP) co-electrospun fiber membranes was measured over a frequency range of 20 Hz to 10^5^ Hz, as shown in Figure 7a. The dielectric constant decreases significantly with increasing frequency, which is attributed to the diminishing contribution of interfacial (Maxwell–Wagner–Sillars) polarization at higher frequencies [29,30]. At low frequencies, dipoles and interfacial charge carriers can effectively respond to the applied electric field. However, as the frequency increases, their relaxation time becomes insufficient to follow the alternating field, resulting in a reduced dielectric response.

For comparison, the dielectric constants at 20 Hz and 1 kHz are summarized in Figure 7d. The dielectric constant of the co-electrospun membranes (M1–M5) decreases with increasing P(VDF-HFP) flow rate. A comparison with the fiber diameters shown in Figure 1 indicates that samples prepared with lower P(VDF-HFP) flow rates exhibit smaller fiber diameters. Thinner fibers provide a higher surface-area-to-volume ratio, which enhances interfacial polarization due to increased charge accumulation at fiber interfaces. Conversely, thicker fibers formed at higher P(VDF-HFP) flow rates reduce the effective interfacial area, resulting in weaker Maxwell–Wagner–Sillars polarization and consequently lower dielectric constant values. Interfacial polarization primarily arises from charge buildup at interfaces between regions with different electrical properties [31]. The interfacial polarization mechanism shows a strong dependence on frequency and tends to disappear as the frequency increases. At very low frequencies, it is evident that the interfacial Maxwell–Wagner effect of the co-electrospun membranes (M1) is higher than pure PVA fibers (M0). Although the pure PVA fibers (M0) possess relatively small diameters (Figure 1a), their dielectric constant remains relatively limited. This behavior can be attributed to the homogeneous nature of the PVA network, where fewer heterointerfaces exist to facilitate charge accumulation. In heterogeneous dielectric materials, interfacial polarization arises from the accumulation of charges at the interfaces between regions with different electrical properties, including interlayer regions. Yang et al. reported that interfacial compatibility refers to the ability of two adjacent phases to interact and adhere effectively at their interface, which plays a crucial role in promoting efficient charge transfer across the interface in composite materials [32].

In Figure 7b, the dielectric loss (tan δ) arises primarily from dipolar relaxation and charge carrier migration within the material [33]. The dielectric loss exhibits a similar trend to that of the dielectric constant, as both parameters are primarily governed by polarization mechanisms. According to a previous study, the conductivity of PVA-based nanocomposites was reported to be approximately 1.6 × 10^−10^ S m^−1^ [34]. As shown in Figure 7c, the conductivity of the samples gradually decreases with increasing P(VDF-HFP) flow rate across M1–M5. As the P(VDF-HFP) fraction increases, the average fiber diameter increases (Figure 1), resulting in a reduced surface area. The decrease in surface area limits interfacial charge accumulation and weakens interfacial polarization effects. In addition, the fluorinated structure of P(VDF-HFP) exhibits more insulating characteristics, which restricts charge carrier mobility. Moisture absorption in PVA-based materials can influence electrical conductivity due to water-induced ionic conduction. As P(VDF-HFP) content increases, the membrane becomes more hydrophobic, as confirmed by the increase in the water contact angle from 77.46° for M0 to 130.95° for M5. This increased hydrophobicity likely reduces water uptake in the membrane, which in turn may suppress water-driven ionic conduction in the PVA phase. Therefore, the reduced moisture absorption with higher P(VDF-HFP) content could contribute to the observed decrease in electrical conductivity. Consequently, the overall conductivity decreases as the P(VDF-HFP) flow rate increases.

### 2.6. Energy Storage Performances

**Figure 8:**
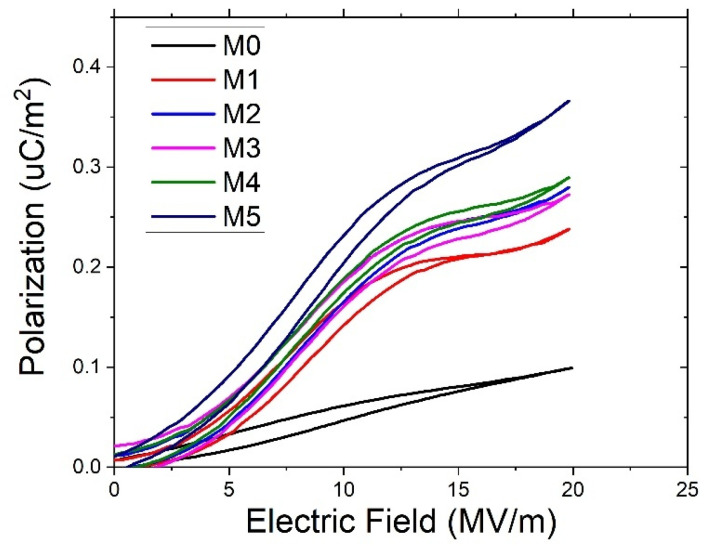
Polarization behavior of PVA/P(VDF-HFP) co-electrospun fiber membranes under different applied electric fields from experiment.

The polarization behavior of the co-electrospun fiber membranes under different electric fields is presented in Figure 8. The polarization increases progressively with increasing P(VDF-HFP) content at the same electric field, indicating an enhanced dipolar response within the co-electrospun fiber membranes. This behavior can be attributed to the combined contribution of interfacial polarization between the two polymer phases and the intrinsic polarization of P(VDF-HFP), particularly its β-phase component [35]. The FTIR analysis (Figure 6) shows that the β-phase fraction increases with increasing P(VDF-HFP) content, which is consistent with the enhanced polarization observed in Figure 8. In addition, for each sample, the polarization increases with increasing electric field as the dipoles progressively align along the field direction.

**Figure 9:**
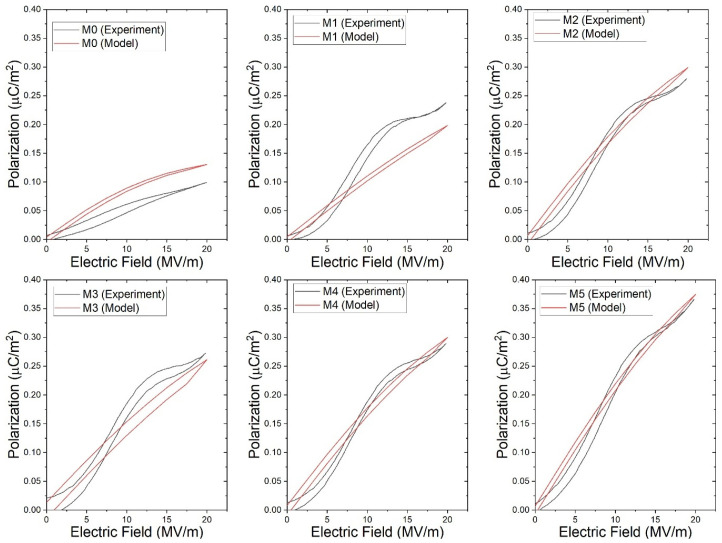
The experimental and modeled polarization behavior of PVA/P(VDF-HFP) co-electrospun fiber membranes under different applied electric fields.

Figure 9 shows the experimental and modeled polarization–electric-field (P–E) curves of the PVA/P(VDF-HFP) co-electrospun fiber membranes (M0–M5). The modeled curves follow a hyperbolic tangent (tanh) function describing dipole switching behavior in dielectric materials. Within the investigated electric field range, the curves remain nearly linear with only slight curvature. Despite this simplified shape, the modeled curves reproduce the overall polarization trend observed experimentally, indicating that the proposed model adequately describes the field-dependent polarization behavior of the co-electrospun membranes.

**Figure 10:**
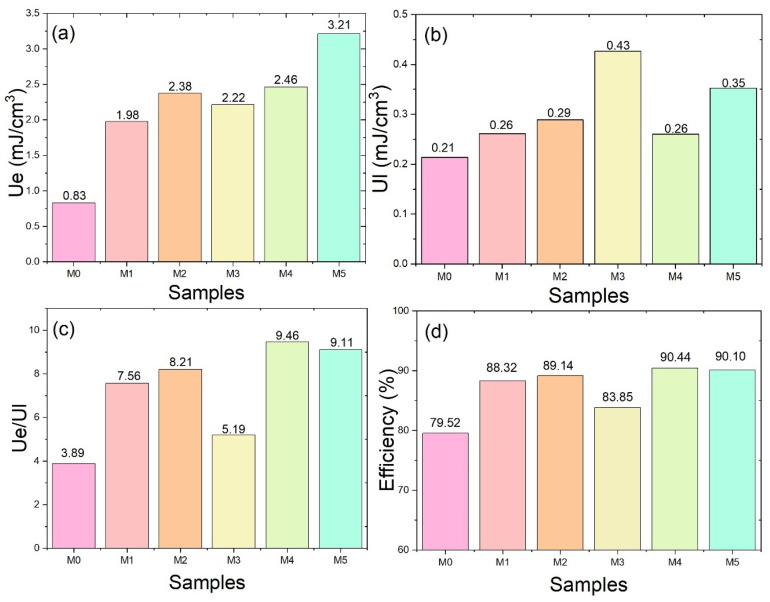
Comparison between experimental and modeled polarization behavior of PVA/P(VDF-HFP) co-electrospun fiber membranes under different applied electric fields: (**a**) energy storage density (Ue), (**b**) energy loss (Ul), (**c**) Ue/Ul ratio, and (**d**) energy storage efficiency.

The recoverable energy storage density (U_e_) and energy loss (U_l_) were calculated from the areas of the P–E hysteresis loops shown in Figure 8. Electrospun fibrous membranes possess a highly porous structure and large surface area, which can lead to localized electric field concentration and surface charge accumulation. These characteristics may induce premature electrical breakdown compared with dense polymer films, making the accurate determination of intrinsic breakdown strength more challenging. The P–E measurements were performed under an applied electric field of 20 MV m^−1^, which corresponds to the maximum stable field that could be applied to the fibrous membranes without electrical breakdown. As shown in Figure 10a, the energy storage densities of M0, M1, M2, M3, M4, and M5 are 0.83, 1.98, 2.38, 2.22, 2.46, and 3.21 mJ cm^−3^, respectively, showing an overall increasing trend. Figure 10b shows a slight increase in the energy loss (U_l_) with increasing P(VDF-HFP) content. Meanwhile, the U_e_/U_l_ ratio in Figure 10c generally increases, indicating an improved balance between energy storage and energy loss. Consequently, the energy efficiency shown in Figure 10d approaches approximately 90% for samples with higher P(VDF-HFP) content. This behavior suggests that although the polarization increases with increasing P(VDF-HFP) content, the dipole alignment becomes more reversible under the applied electric field, which helps limit hysteresis loss. As a result, a larger fraction of the stored energy can be recovered, leading to improved energy storage efficiency. The progressive increase in U_e_ with increasing P(VDF-HFP) content is consistent with the enhanced polarization observed in Figure 8. In particular, the highest energy storage density obtained for M5 is attributed to its greater polarization capability and improved dipole alignment under the applied electric field.

Although the dielectric constant decreases with increasing P(VDF-HFP) content, which is achieved by increasing the P(VDF-HFP) flow rate during electrospinning, the energy storage density increases. This difference arises from the distinct mechanisms governing dielectric permittivity and ferroelectric energy storage. The dielectric constant measured by the LCR meter is largely influenced by interfacial polarization within the fibrous structure. As the P(VDF-HFP) flow rate increases, the fiber diameter becomes larger, leading to a reduced effective interfacial area and consequently weakening interfacial polarization. In contrast, the energy storage behavior is mainly determined by ferroelectric polarization associated with the crystalline phase structure of P(VDF-HFP). The increased β-phase fraction promotes stronger dipole alignment under the applied electric field, leading to enhanced polarization and higher recoverable energy storage density.

Furthermore, the energy efficiency (Figure 10d), derived from the ratio of energy storage density to total energy input, reflects the balance between polarization and dielectric loss. The relatively high efficiency values observed for samples with higher P(VDF-HFP) content suggest that the increased polarization does not lead to excessive energy dissipation, making these co-electrospun membranes promising for dielectric energy storage applications. From Table 1, the energy storage performance of previously reported polymer-based dielectric materials is summarized. Although higher energy storage densities are often achieved at elevated electric fields, the co-electrospun PVA/P(VDF-HFP) membranes in this work exhibit relatively high energy efficiency at a low electric field (≈90% at 20 MV m^−1^), indicating their suitability for efficient low-field energy storage applications.

## 3. Materials and Methods

The PVA solution was loaded into a 20 mL plastic syringe equipped with a stainless-steel needle and delivered using a syringe pump (Nz1000, New Era Pump Systems, Toledo St Farmingdale, NY, USA) at a constant flow rate of 1.0 mL h^−1^. The flow rate of the P(VDF-HFP) solution was varied at 1.0 (M1), 2.0 (M2), 3.0 (M3), 4.0 (M4), and 5.0 (M5) mL h^−1^. The collector was rotated at a constant speed of 300 rpm. A high-voltage DC power supply was used to generate the electric field. The applied voltages for the PVA and P(VDF-HFP) solutions were 18 and 14 kV, respectively, with the positive electrode connected to the stainless-steel needle. Since the total deposited volumes of PVA and P(VDF-HFP) at the collector were approximately equivalent, their resulting layer thicknesses were assumed to be comparable. The thickness of the co-electrospun fiber membranes was systematically controlled by varying the electrospinning duration. All membranes exhibited a thickness of approximately 320 ± 30 μm. Following fabrication, the membranes were dried at 120 °C for 30 min to eliminate residual solvent.

**Figure 11:**
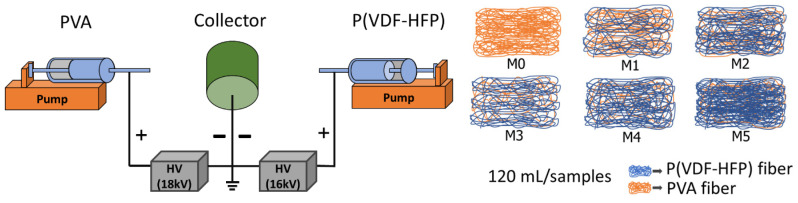
Schematic diagram of the electrospinning setup for the fabrication of PVA and P(VDF-HFP) co-electrospun fiber membranes.

Co-electrospun fiber membranes were fabricated via the electrospinning process, as illustrated in Figure 11. Polyvinyl alcohol (PVA), purchased from Chem-Supply Pty Ltd. (Gillman, Australia), was dissolved in deionized (DI) water at a weight-to-volume ratio of 1:7 (*w*/*v*). Polyvinylidene fluoride–hexafluoropropylene (P(VDF-HFP)) powder (Solef 11010/1001), obtained from Solvay Solexis (Brussels, Belgium), was dissolved in N,N-dimethylformamide (DMF) at a weight-to-volume ratio of 1:3 (*w*/*v*). The PVA solution was magnetically stirred at 50 °C and 200 rpm for 6 h, whereas the P(VDF-HFP) solution was stirred under identical conditions for 4 h to ensure complete dissolution and homogeneity. Subsequently, the prepared solutions were maintained at 35 °C for 2 h to eliminate entrapped air bubbles prior to electrospinning.

### 3.1. Physical Characterization

#### 3.1.1. Scanning Electron Microscopy (SEM)

The morphologies of the PVA and P(VDF-HFP) co-electrospun fibers were characterized using scanning electron microscopy (SEM; TM3030Plus, Hitachi, Tokyo, Japan). Fiber diameters were subsequently determined from the SEM micrographs using ImageJ software, version 1.53k. The diameter distribution histogram was constructed from measurements of at least 100 individual fibers per sample, where the diameter of each fiber was measured and counted as one data point to determine the frequency of fibers within each diameter range.

#### 3.1.2. Contact Angle

Surface wettability was characterized using static water contact angle measurements. In each measurement, a 3 μL deionized water droplet was gently placed on the sample surface, and the contact angle was determined from the droplet profile. The wetting state was analyzed according to the Wenzel and Cassie–Baxter models [19,39]. The former describes homogeneous wetting with complete liquid infiltration into surface asperities, while the latter represents a composite interface formed by entrapped air beneath the droplet.

#### 3.1.3. X-Ray Diffraction (XRD)

The crystalline structures of the co-electrospun fiber membranes were characterized by X-ray diffraction (XRD; PANalytical Empyrean, Almelo, The Netherlands). XRD measurements were conducted to identify the crystalline phases present in all samples. The diffraction patterns were recorded at a scanning rate of 0.05° s^−1^ using Cu Kα_1_ radiation (λ = 0.15406 nm) operated at 40 kV and 30 mA. The resulting patterns were compared with standard reference data for phase identification. The crystallite size of PVA was estimated from the (101) reflection at 2θ ≈ 19.5°, while the crystallite size of P(VDF-HFP) was calculated using the β-phase (110)/(200) reflection at 2θ ≈ 20.0°. These peak assignments were based on previously reported literature [21,22,23,24]. In addition, the crystallite size (D) was estimated using the Scherrer equation, as shown in Equation (1).

(1) $$D=\frac{K\lambda }{\beta \cos\theta }$$

where K is the Scherrer constant, λ is the wavelength of the X-ray radiation, β is the Full width at half maximum (FWHM) of the peak, and θ is the Bragg angle. The Scherrer constant (K) is a shape factor, typically assumed to be 0.9 [40]. The FWHM (β) used in the calculation was corrected for instrumental broadening according to

(2) $$\beta =\sqrt{\beta_{m}^{2}-\beta_{i}^{2}}$$

where $\beta_{m}$ and $\beta_{i}$ are the measured peak broadening and instrumental broadening, respectively.

#### 3.1.4. Fourier-Transform Infrared Spectroscopy (FTIR)

The functional groups of the PVA and P(VDF-HFP) co-electrospun fiber membranes were characterized using Fourier-transform infrared spectroscopy (FTIR; Vertex 70, Bruker, Germany). The spectra were recorded in transmission mode over the wavenumber range of 400–4000 cm^−1^ with a resolution of 4 cm^−1^. The transmittance data were converted to absorbance for further analysis. The characteristic absorption bands of the constituent polymers were identified and compared with standard reference data for functional group assignment.

Furthermore, the relative fraction of the β-phase, F(β), was calculated from the absorbance values at 764 cm^−1^ (α-phase) and 840 cm^−1^ (β-phase) according to the following equation:

(3) $$F\left(\beta \right)\%=\frac{A_{\beta }}{\left(\frac{\kappa_{\beta }}{\kappa_{\alpha }}\times A_{\alpha }\right)\;+\;A_{\beta }}\times 100$$

where Aα and Aβ denote the absorbance values at 764 and 840 cm^−1^, corresponding to the α and β crystalline phases of P(VDF-HFP), respectively. Here, κ_α_ and κ_β_ denote the absorption coefficients of the α and β phases, with values of 6.1 × 10^4^ and 7.7 × 10^4^ cm^2^ mol^−1^, respectively [41,42,43].

### 3.2. Dielectric Properties

The dielectric properties of the samples were measured using an LCR meter (IM3533, HIOKI, Nagano, Japan) over a frequency range of 20–10^5^ Hz at room temperature. The electrospun fibrous membranes were tested in a parallel-plate configuration, in which the sample was placed on the bottom electrode and contacted by the upper probe electrode to establish electrical contact during the measurement. Before measurement, open and short compensation procedures were performed to minimize the influence of parasitic impedance from the measurement system. Since all samples were measured under the same experimental conditions, the calculated dielectric constant mainly reflects the relative differences among the samples.

The dielectric constant (ε_r_) and electrical conductivity (σ) were calculated from the measured capacitance and conductance values according to Equations (4) and (5), respectively [44,45,46].

(4) $$\varepsilon_{r}=\frac{Cd}{\varepsilon_{0}A}$$

(5) $$\sigma =\frac{Gd}{A}$$

where *C* is the capacitance (F), d is the thickness of the membrane (m), $\varepsilon_{0}$ is the permittivity of vacuum (8.854 × 10^−12^ F m^−1^), *A* is the electrode area (m^2^), and *G* is the conductance (S).

### 3.3. Energy Storage Properties

The ferroelectric properties of the samples were characterized using a P–E Loop Ferroelectric Test System (PK-CPE1701, PolyK Technologies, Philipsburg, PA, USA). When the applied electric field (E) varies between two equal but opposite values, the polarization (P) approaches the spontaneous polarization ($P_{s}$) at sufficiently high electric fields. At zero electric field, a nonzero polarization remains, defined as the remnant polarization ($P_{R}$), whereas the polarization becomes zero at the coercive field ($E_{C}$).

The measured P–E hysteresis loops exhibit symmetric switching behavior, which is consistent with the phenomenological hyperbolic tangent (tanh) model commonly used to describe saturated ferroelectric loops. In this model, the polarization branches can be expressed as follows:

(6) $$P^{+}\left(E\right)=P_{s}\tanh\left[\frac{E-E_{C}}{2d}\right]$$

(7) $$P^{-}\left(E\right)=-P^{+}\left(-E\right)$$

where the parameter *d* is related to the switching characteristics of the loop and is theoretically defined as:

(8) $$d=E_{C}{\left[log\left(\frac{1+P_{R}/P_{s}}{1-P_{R}/P_{s}}\right)\right]}^{-1}$$

Here, $P_{s}$, $P_{R}$, and $E_{C}$ denote the spontaneous polarization, remnant polarization, and coercive field, respectively [47].

The energy storage density ($U_{e}$) and energy efficiency (η) were determined from the area enclosed by the P–E hysteresis loop. The recoverable energy density is calculated as [48,49]:

(9) $$U_{e}=\int_{P_{R}}^{P_{s}}EdP$$

The energy efficiency is given by

(10) $$\eta \left(\%\right)=\frac{U_{e}}{U_{e}+U_{l}}\times 100\%$$

where $U_{l}$ represents the energy loss density associated with the hysteresis loop.

## 4. Conclusions

This work investigated the dielectric and ferroelectric behavior of PVA/P(VDF-HFP) co-electrospun fiber membranes prepared via electrospinning. The dielectric constant of the co-electrospun membranes (M1–M5) decreased with increasing P(VDF-HFP) flow rate, which is closely related to the variation in fiber diameter and the corresponding interfacial polarization. Relatively finer P(VDF-HFP) fibers formed at lower flow rates provided a larger surface-area-to-volume ratio, thereby enhancing interfacial charge accumulation and resulting in a higher dielectric constant. SEM analysis revealed systematic changes in fiber diameter and morphology with increasing P(VDF-HFP) flow rate, which directly influenced interfacial polarization. In contrast, M0 exhibits a lower dielectric constant despite having relatively small fiber diameters, since it consists only of PVA and lacks polymer–polymer interfaces that promote interfacial polarization. As a result of the enhanced ferroelectric polarization, the energy storage density of the PVA/P(VDF-HFP) co-electrospun fiber membranes increased from 0.83 mJ cm^−3^ (M0) to 3.21 mJ cm^−3^ (M5) due to the significantly enhanced β-phase content. The increased β-phase fraction was confirmed by FTIR analysis through the strengthened absorption band at 840 cm^−1^ and further supported by the corresponding β-phase diffraction peak observed in XRD. These findings demonstrate that the dielectric characteristics and molecular polarization of the co-electrospun membranes play a crucial role in determining their energy storage performance, indicating their potential for low-field flexible energy storage applications with high energy efficiency.

## Figures and Tables

**Table 1:** Comparison of energy storage performance among different co-electrospun fiber mem-brane samples.

Samples	Method	Ue (mJ/cm^3^)	Efficiency (%)	Ref.
P(VDF-HFP)	Solvent casting	4500 @350 MV/m	70 @350 MV/m	[36]
P(VDF-HFP)	Electrospinning and hot-pressing	7000 @350 MV/m	30 @350 MV/m	[35]
P(VDF-HFP)	Electrospinning and hot-pressing	2000 @250 MV/m	70 @250 MV/m	[37]
PVDF-HFP/PiBMA	Electrospinning and hot-pressing	1250 @120 MV/m	80 @120 MV/m	[38]
PVDF-HFP/PVDF-TrFE	Electrospinning and hot-pressing	500 @120 MV/m	73 @120 MV/m	[38]
PVA/P(VDF-HFP) (M5)	Electrospinning	3.21 @20 MV/m	90 @20 MV/m	This work

## Data Availability

The data presented in this study are available from the corresponding authors upon reasonable request.
